# Clinical Documentation and Data Transfer from Ebola and Marburg Virus Disease Wards in Outbreak Settings: Health Care Workers’ Experiences and Preferences

**DOI:** 10.3390/v6020927

**Published:** 2014-02-19

**Authors:** Silja Bühler, Paul Roddy, Ellen Nolte, Matthias Borchert

**Affiliations:** 1 London School of Hygiene and Tropical Medicine, Keppel Street, London WC1E 7HT, UK; E-Mails: enolte@rand.org (E.N.); Matthias.Borchert@charite.de (M.B.); 2 Institute of Social and Preventive Medicine, University of Zurich, Hirschengraben 84, Zurich 8001, Switzerland; 3 Médecins Sans Frontières – Spain, Nou de la Rambla, 26, Barcelona 08001, Spain; E-Mail: paul.roddy@barcelona.msf.org; 4 RAND Europe, Westbrook Centre, Milton Road, Cambridge, CB4 1YG, UK; 5 Institute of Tropical Medicine and International Health, Charité – Universitätsmedizin Berlin, Spandauer Damm 130, Berlin D-14050, Germany

**Keywords:** viral hemorrhagic fever, Ebola hemorrhagic fever, Marburg hemorrhagic fever, Ebola virus disease, Marburg virus disease, isolation wards, clinical documentation, data transfer

## Abstract

Understanding human filovirus hemorrhagic fever (FHF) clinical manifestations and evaluating treatment strategies require the collection of clinical data in outbreak settings, where clinical documentation has been limited. Currently, no consensus among filovirus outbreak-response organisations guides best practice for clinical documentation and data transfer. Semi-structured interviews were conducted with health care workers (HCWs) involved in FHF outbreaks in sub-Saharan Africa, and with HCWs experienced in documenting and transferring data from high-risk areas (isolation wards or biosafety level 4 laboratories). Methods for data documentation and transfer were identified, described in detail and categorised by requirement for electricity and ranked by interviewee preference. Some methods involve removing paperwork and other objects from the filovirus disease ward without disinfection. We believe that if done properly, these methods are reasonably safe for certain settings. However, alternative methods avoiding the removal of objects, or involving the removal of paperwork or objects after non-damaging disinfection, are available. These methods are not only safer, they are also perceived as safer and likely more acceptable to health workers and members of the community. The use of standardised clinical forms is overdue. Experiments with by sunlight disinfection should continue, and non-damaging disinfection of impregnated paper, suitable tablet computers and underwater cameras should be evaluated under field conditions.

## 1. Introduction

Filoviruses, *i.e*., marburgviruses and ebolaviruses, are highly infectious and transmitted from person-to-person by direct contact with infected body fluids or by contaminated fomites [1,2]. The case fatality ratios of filovirus hemorrhagic fevers range from 25% to 90% [1]. In order to better understand human clinical manifestations of FHF and to inform treatment strategies, there is a need to systematically collect clinical data during outbreaks. 

FHF outbreaks usually attract international response teams, who bring additional staff and substantial amounts of equipment and supplies to the field. Nevertheless, clinical documentation inside filovirus disease wards during FHF outbreaks has been limited [3,4,5,6,7,8]. Data collection has not always been systematic, and data have been lost as clinical records considered to be contaminated were destroyed. There is a lack of guidelines or standardised procedures for documenting clinical FHF data and transferring them from the FHF ward to the outside, and no consensus on the safest or easiest methods. 

This study aims to contribute to our understanding of potential approaches for documenting and transferring clinical FHF data by conducting a survey among health care workers with relevant experience.

## 2. Methods

We conducted semi-structured interviews with HCWs involved in FHF outbreaks in sub-Saharan Africa, and with HCWs experienced in documenting and transferring data from high-risk areas (isolation wards or biosafety level 4 (BSL-4) laboratories). We used snowball sampling, starting with a list of HCWs personally known by two researchers on the team (PR, MB) to have filovirus experience. Filovirus-experienced HCWs were affiliated with non-governmental organisations, public health institutions responsible for disease control, and academic institutions. Interviewees with experience in FHF outbreaks in Kikwit, Democratic Republic of Congo (COD) (1995), Durba, COD (1998/1999), Gulu, Uganda (2000/2001), Uíge, Angola (2004/2005), Kasai, COD (2007), Bundibugyo, Uganda (2007) had participated in epidemiological investigations, provision of health care, project coordination, training of local staff, organisation of filovirus disease ward, data management, *etc*.

Interviews sought to understand: (1) Reasons for not collecting or obtaining clinical data, (2) Methods employed to date for data collection and transfer, (3) Quality criteria for methods, (4) Advantages and disadvantages of methods used to date, (5) Possible improvements for existing methods, and (6) Possible new methods for data collection. Telephone interviews were conducted by the lead author and lasted on average 15 min. Interviews were performed using an interview guide (Supplementary Material), were audio recorded and transcribed verbatim. Data were analysed using qualitative research methods, *i.e*., a structured extraction template, organised in line with the themes guiding the interview described above. 

Suggestions from the first round of interviews were presented to a subgroup of interviewees who had been involved in FHF outbreaks in sub-Saharan Africa, because their views on the practicality of the suggestions were deemed to be particularly relevant. They were invited to rank by preference methods of data documentation and transfer identified in the first round. Methods were grouped according to whether or not their application required electrical power in the filovirus disease ward. 

Verbal informed consent for participation in the interview process was obtained from all participants prior to the interview. Ethics approval was granted by the Ethics Committee of the London School of Hygiene and Tropical Medicine.

## 3. Results

Forty HCWs with experience in FHF outbreaks or data documentation and transfer from BSL-4 wards or isolation wards representing 15 organisations were initially contacted via e-mail for the first round of interviews. Of these, 30 individuals (75%) from 14 organisations (93%) responded, and 21 HCW (53%) from 13 organisations (87%) agreed to be interviewed [9]. For the second round we contacted 16 interviewees; eight (50%) responded and agreed to be interviewed again. 

### 3.1. Clinical Documentation inside Filovirus Disease Wards

Clinical documentation was reported to be often unsystematic and haphazard. While predominantly identified as important, many interviewees involved in FHF outbreaks did not believe that clinical documentation was a top priority. The importance of clinical documentation in outbreak situations has increased in HCWs’ perceptions in recent years; however staff safety, patient clinical care, and outbreak containment still ranked higher. High temperatures and the obligatory use of personal protective equipment were perceived to make it difficult to remain in a filovirus disease ward longer than necessary for providing care. Many interviewees felt overwhelmed by high patient numbers, particularly at the beginning of an outbreak. Filovirus disease ward responsibilities were often carried out by staff without prior FHF experience, who might not always appreciate the particular importance of clinical documentation in the context of FHF. Respondents with experience in filovirus outbreaks diverged on the issue of data ownership, with some believing these to belong to the hospital, while others thought that these should be owned by the organisation supervising the filovirus disease ward and collecting the data. 

Interviewees noted that to prevent their potential contamination, clinical records were kept separate from the patient and handled only after gloves had been disinfected with chlorine solution. On occasion, the outer pair of gloves was removed before handling the records. Nevertheless, clinical data were frequently destroyed intentionally, out of concern of contamination:

“Almost all of these data were eventually destroyed [when the paper-based clinical records] were sprayed with chlorine” (note: chlorine solution destroys paper-based records).

### 3.2. Data Transfer from Filovirus Disease Wards

#### 3.2.1. Data Transfer Methods from Sub-Saharan Africa

Fourteen of the 16 HCWs involved in FHF outbreaks had transferred data or had witnessed data being transferred out of filovirus disease wards. Data transfer was generally perceived as difficult, and no method currently used as quite satisfactory. Quality criteria for data transfer methods were safety, practicability, timeliness; readability, accuracy and comprehensiveness of the data; acceptability by filovirus disease ward clinicians and staff. 

A range of methods have been used in FHF outbreak settings:

- Some methods avoided taking any objects out of the filovirus disease ward: Clinical notes were recorded by memory once outside the ward (n = 6 interviewees); clinical notes were dictated to a HCW on the other side of the fence (6); clinical notes were held up at the fence and then photographed (5), manually copied (2), or entered into a laptop computer (1) by a HCW standing outside the fence.
- Other methods avoided taking paper forms out of the filovirus disease ward but involved taking other objects out after disinfection: Patient records were photographed inside the ward with a digital camera wrapped in a plastic bag, which was disinfected by chlorine before taking it out (1); a designated laptop was used inside the ward for data entry, sprayed with chlorine solution, and taken out at the end of the day (1).
- Again other methods involved taking paperwork out of the filovirus disease ward: without disinfection (5), after exposing it to sun light (UV radiation; 1) or after disinfecting it with chlorine solution after wrapping it in plastic (1) or without doing so (1); data were copied from patient files into forms without touching anything else, and these forms were then taken out without disinfection (1); ward rounds were conducted by two HCWs, one providing patient care, the other taking clinical notes without touching anything on the ward so that records were considered uncontaminated and taken out without disinfection (2); following a patient’s death, the single-patient room including the patient records was sealed and fumigated with formaldehyde, the records then taken out (1).

#### 3.2.2. Methods used in BSL-4 Laboratories in Europe

Methods included data transfer via fax, Internet, telephone, or voice activated dictation machine. In developed countries no paperwork was removed from isolation areas. When patients are treated inside individual patient isolators, paperwork is considered uncontaminated and handled without further procedure. 

#### 3.2.3. Suggested Methods for the Future

Interviewees suggested a range of methods for data collection in future filovirus outbreak settings:

- Enter data or scan records into a laptop computer left inside, and transmit data via Internet, a cable, or a USB stick disinfected with chlorine to another computer outside (n = 8 interviewees)
- Have a person entering the filovirus disease ward and photographing the clinical records without touching anything else and taking the camera out without disinfection (5)
- Enter data with a Personal Digital Assistant (PDA) kept inside a plastic cover, disinfect it with chlorine before taking it out, or transmit data via Bluetooth or email (3)
- Make carbon copies or photocopies of the clinical records, wrap them in a plastic cover, disinfect the cover and take only the copies out (2)
- Print patient forms on transparencies, use a permanent marker to fill them out and spray them with chlorine (2).
- Use a voice recorder inside the ward and transfer the audio cassette or minidisc to the outside (2)
- Use a walkie-talkie or a cell phone to dictate clinical data to the outside (2)
- Transmit data with a fax machine inside connected via cable to another fax machine outside (2)
- Place documents in a container after the outbreak, leave them inside until the virus is considered unviable, or fumigate the container with formaldehyde (1)
- Have a video camera pointing at the table where data are documented (1)

### 3.3. Perceived Advantages and Disadvantages of Present and Future Methods

#### 3.3.1. General Aspects

Actual and perceived safety was a general concern about taking material out of a filovirus disease ward. Although most interviewees believed it safe to take paperwork out if not visibly soiled with body fluids, residual, occasionally irrational doubt surrounded this issue, as illustrated in some of the verbatim quotes. Chlorination, fumigation or other methods of disinfection would not make all interviewees feel sufficiently safe about taking potentially contaminated objects out of the filovirus disease ward:

“Who knows if there are odd chances and somebody might get Ebola from a microscopic bit of virus somewhere that escaped notice”.

(HCW 15)

Another aspect was the safety as perceived by the community: even effective disinfection may not prevent concerns about spreading the disease into the community by taking objects out of the filovirus disease ward, particularly if new cases happen to emerge in the community. There was a notion that such ‘rumours’ might easily lead to accusations against those supervising the ward, and should best be avoided:

“If you take something out of the isolation ward and then the epidemic flairs up in the community again, [there would be trouble]. I mean that is an environment where there are a lot of rumours and perhaps … the risk is not really there, but you don’t even want to be seen as taking risks”.

(HCW 14)

Technical solutions for data transfer not requiring the removal of objects from the filovirus disease ward were unanimously considered to be safe. They were also thought to be practical, as data would be ready to be analysed. Lack of Internet connectivity, breakdown of equipment due to heat or humidity or loss through theft might be challenges. There was disagreement about the implications of required power. In remote areas of sub-Saharan Africa, where most outbreaks occur, electricity is often not readily available, or, if so, it may not be accessible on the filovirus disease ward itself.

#### 3.3.2. Detailed Advantages and Disadvantages of Methods

HCWs mentioned the following experienced advantages and disadvantages of methods used in past FHF outbreaks: Writing down clinical data from memory outside the ward is safe and allows full concentration on clinical work inside the ward, but the accuracy of recalled data is likely to be poor. Spraying paperwork with chlorine can hamper its readability. Dictating over the fence is time-consuming, tiresome when standing in the sun in full protective gear, and can result in erroneous recordings. Fumigating rooms at the end of an outbreak is quick and leaves paperwork intact, but the substance is toxic, and fumigation, like other methods of disinfection, does not provide proof that the virus is unviable. Using a tape recorder is easy, but requires an algorithm to collect data in a standardised way. Using a camera is quick and easy for non-medical staff, but data entry from pictures for statistical analysis is cumbersome. Laptops sprayed with chlorine should not be expected to last long.

HCWs pointed out the following anticipated advantages and disadvantages of new methods suggested for future outbreaks: When a voice recorder is used inside the ward it may be damaged by spraying it with chlorine solution before taking it out. A fax or scanner would allow capturing the information of the whole patient file, including notes that “the clinician has scribbled on the margin” (HCW 14). PDAs require a standardized data entry mask and were therefore considered as too technologically demanding by some, while others felt that using PDAs would be practical and relatively inexpensive. 

Nearly all interviewees called for flexibility in choosing the method for documenting and transferring clinical data:

“At the very beginning you don’t have … many resources and you might want to use … a very simple method … and as your team increases … you could do more sophisticated things”.

(HCW 2)

### 3.4. Ranking of Methods

Interviewees involved in FHF outbreaks ranked existing and suggested methods as follows: Among the methods not requiring electricity, the one that ranked highest was ‘entering the filovirus ward with two HCWs, one providing care to the patient, the other documenting clinical data’. If the analysis of clinical data is urgent during an outbreak, ‘dictating over the fence’ was ranked even higher. Among methods requiring electricity in the field but not on the ward, ‘using a PDA in a waterproof bag which is disinfected and taken outside’ ranked highest. Among methods requiring electricity on the filovirus disease ward, ‘keeping a PDA on the filovirus ward that transmits data via Bluetooth or email’ was preferred.

## 4. Discussion

Only a limited amount of clinical data from FHF outbreaks has been collected and preserved [4]. By presenting methods of data collection and transfer experienced by health care workers having contributed to the response to filovirus disease outbreaks we wish to raise attention to this overlooked problem. We are convinced that this problem exists in filovirus disease outbreaks, but it may also occur during disease outbreaks due to other highly pathogenic viruses, such as Lassa, Lujo, Bas-Congo, Machupo or Crimean-Congo hemorrhagic fever virus.

Most interviewees agreed that clinical documentation is a “neglected issue”. A primary reason for the scarcity of human filovirus clinical data was that an interest in clinical documentation during FHF outbreaks has only developed in recent years among HCWs, and still competes with other priorities in an outbreak situation. 

Clinical documentation of FHF infections had not been standardised in past outbreaks, while a standard form has only been recently developed [5]. Methods for documenting and transferring clinical data used until now were perceived as problematic, but several suggestions for future improvement were made, with a clear ranking emerging. 

The top-ranked method not requiring electricity was ‘going inside the filovirus ward with two HCW, one conducts patient consultations, while another collects patient clinical data. This could be an appropriate method, with the advantage that the first HCW can concentrate on clinical duties and does not need to spend additional time in the ward for data collection. It is anyway good practice that the HCW providing clinical care is accompanied by a second HCW ensuring that safety procedures are followed (*i.e*., a “buddy” approach). This second HCW could also be responsible for clinical data collection. We believe that another viable solution may be a HCW entering the ward regularly to copy patient files lying open on a table, without touching anything else. The copies could be taken out of the filovirus disease ward without disinfection with the original paperwork remaining inside. The copies could later be taken elsewhere and be analysed by the organisation supervising the FHF ward, subject to data sharing agreements with authorities of the host country. Further copies could be produced for the host country’s authorities. The originals would remain on the ward, available to those providing clinical care, and would be destroyed at the end of the outbreak.

The safety of the methods described above depends on HCW’s ability to avoid accidental contamination of the paperwork they later take out without disinfection, and on the viability of filoviruses on contaminated paperwork. Sagripanti *et al.* have carried out experiments with simulated sunlight in BSL4 laboratories, exposing Ebola virus to UV light “for selected times up to 30 s” [10]. 3%–4% of the Ebola virus population survived due to virus being shielded in cellular debris. Although this is a minor fraction, it constitutes a safety risk. HCW’s ability to avoid accidental contamination of paperwork depends on individual factors, but also environmental ones like workload, lighting, crowding *etc*. In many, but not all situations, methods described above that aim at reducing the risk of accidental contamination to a minimum without ruling it out entirely may be reasonably safe, but it is difficult to recommend them in general: whether a method involving removing paperwork from the filovirus disease ward without disinfection is sufficiently safe must be decided on a case-by-case basis. Safety could be improved by using impregnated paper that survives treatment with disinfectant [11]. Data documentation forms could be printed on such paper and filled out by using a ballpoint. 

Among methods requiring electricity in the field but not on the filovirus disease ward, ‘having a PDA in a plastic bag and taking it outside after disinfection’ ranked highest. As discussed for paperwork using PDAs or successor technology like tablet computers and smartphones without disinfection can be considered as reasonably safe and feasible in certain settings. An improvement over hardware protected by a plastic bag may be the use of a suitable tablet PC (Toughbook CF-H1 Mobile Clinical Assistant, Panasonic) as described by Bente *et al.* for a BSL4 laboratory, which allows “wiping down with alcohol or quaternary detergents” and may “remain functional after BSL4 decontamination procedures such as formaldehyde fumigation or vaporized hydrogen peroxide treatment” [12]. Possible constraints include staff being unfamiliar with the technology, insufficient time for training during an outbreak, a technical break down under the harsh working conditions, and technicians too busy with other priorities or lacking the skills and tools to repair information technology equipment. We believe that a more practical and reasonably safe solution would be a HCW photographing patient records lying open on a table in the ward without touching anything else, and taking the camera out without disinfection. This method is not technically demanding, and an image of the patient file would include information noted on the margins. Once outside, the pictures could be uploaded on a computer and, if possible, printed out for data entry and analysis. Safety could be enhanced by using an underwater (dive) camera that can be dunked in disinfectant (e.g., Micro-Chem Plus Detergent Disinfectant Cleaner, National Chemical Laboratories, Inc. Philadelphia, PA, USA).

Among methods requiring electricity inside the filovirus disease ward, a ‘PDA transmitting data to a computer outside via Bluetooth or email’ received the highest score. However, technological solutions based on PDA/tablet computer/smartphone, Bluetooth and Internet may be too vulnerable for many outbreak settings in rural sub-Saharan Africa. We believe a scanner transmitting the image of a patient record to a computer outside would be quick, inexpensive, easy to handle, and technically less challenging. 

The use of battery powered respirators on filovirus disease wards has not been experienced or suggested by HCWs in our interviews, but has been discussed before. Although at first sight they may appear to provide some advantages, their use has been dismissed by those who usually run the filovirus disease wards. Arguments against respirators include: (i) whether respirators would be sufficiently comfortable for a prolonged use in the tropical climate is questionable; (ii) it would be logistically difficult to provide them to all potentially exposed health workers in- and outside filovirus disease wards; (iii) sustainability for the period following the international response would be extremely difficult to achieve; (iv) most importantly, they would likely foster the mis-belief that respirators are essential to care for Ebola and Marburg virus disease patients, and significant difficulties to recruit sufficient local and expatriate staff may be the consequence when respirators are not available for everybody.

Differences between outbreak settings must be taken into account: in an urban setting electricity, mobile phone coverage, and Internet may be available, whereas in a remote rural area, they may be non-existent and hard to provide. While we consider all methods discussed above to be reasonably safe—albeit not fool proof—in specific settings, methods which do not require taking objects out of the ward without disinfecting them may have the additional advantage of allaying safety concerns in the community. Given the tendency of FHF outbreaks to occur in resource-poor countries, some solutions will in practice depend on the involvement of an international outbreak response team and the equipment it usually provides, and will hardly help local HCWs recording clinical data while treating patients prior to the arrival of such a team. 

The number of organisations and individuals with profound experience of handling filoviruses is limited. Our survey had a high response rate of 87% (13/15) at the organisational level, while the response rate was still 53% (21/40) at the individual level. Opinions of those who participated may differ from those who did not. 

## 5. Recommendations and Conclusions

Given its free availability, the safety of using sunlight to inactivate filoviruses on paperwork by its UV component and exsiccation should be evaluated further with longer UV light exposure periods mimicking the presence of sun light in most outbreak settings in sub-Saharan Africa. If results are promising, a standard procedure for sunlight disinfection should be developed. 

Using a standardised form [5] in future outbreaks is crucial for improving the documentation of clinical FHF data. These data can then be transferred outside the filovirus disease ward, using one of the methods discussed above. Printing these standardised forms on impregnated paper [11], which can be disinfected before taking it out of the ward, should have a top priority for evaluation in a future outbreak. When preference is given to data transfer via tablet computer or camera, models should be chosen that allow disinfection. In outbreak settings with available electricity the use of scanners should be tested. 

We agree with our interviewees that safety of staff, clinical care of patients, and outbreak containment are top priorities. But we also believe that proper clinical documentation is crucial for advancing our comprehension of poorly understood diseases like FHF, and for ultimately improving the clinical care of FHF patients. Resources assuring clinical documentation should be made available at the initiation of outbreak response. Those with safety concerns should consider a study by Bausch *et al*., who have tested various clinical and environmental specimens from an Ebola disease ward for the presence of the virus and who concluded that “the risk of transmission from fomites in an isolation ward ... is low when currently recommended infection control guidelines for the viral hemorrhagic fevers are followed” [13].

## Supplementary Files

Supplementary File 1 Supplementary Information (PDF, 183 KB)

## References

[1] Heymann D.L. (2004). Control of Communicable Diseases Manual.

[2] Dowell S.F., Mukunu R., Ksiazek T.G., Khan A.S., Rollin P.E., Peters C.J. (1999). Transmission of Ebola hemorrhagic fever: A study of risk factors in family members, Kikwit, Democratic Republic of the Congo, 1995. Commission de Lutte contre les Epidemies a Kikwit. J. Infect. Dis..

[3] Médecins Sans Frontières (2005). Marburg hemorrhagic Fever outbreak. Uige-Angola April–July 2005 MSF OCBA.

[4] Bausch D.G., Sprecher A.G., Jeffs B., Boumandouki P. (2008). Treatment of Marburg and Ebola hemorrhagic fevers: A strategy for testing new drugs and vaccines under outbreak conditions. Antivir. Res..

[5] Colebunders R., Tshomba A., van Kerkhove M.D., Bausch D.G., Campbell P., Libande M., Pirard P., Tshioko F., Mardel S., Mulangu S. (2007). Marburg hemorrhagic fever in Durba and Watsa, Democratic Republic of the Congo: Clinical documentation, features of illness, and treatment. J. Infect. Dis..

[6] Jeffs B., Roddy P., Weatherill D., de la Rosa O., Dorion C., Iscla M., Grovas I., Palma P.P., Villa L., Bernal O. (2007). The Medecins Sans Frontieres intervention in the Marburg hemorrhagic fever epidemic, Uige, Angola, 2005. I. Lessons learned in the hospital. J. Infect. Dis..

[7] Guimard Y., Bwaka M.A., Colebunders R., Calain P., Massamba M., De Roo A., Mupapa K.D., Kibadi K., Kuvula K.J., Ndaberey D.E. (1999). Organization of patient care during the Ebola hemorrhagic fever epidemic in Kikwit, Democratic Republic of the Congo, 1995. J. Infect. Dis..

[8] Roddy P., Colebunders J., Jeffs B., Palma P.P., van Herp M., Borchert M. (2011). Filovirus hemorrhagic fever outbreak case management: A review of current and future treatment options. J. Infect. Dis..

[B9-viruses-06-00927] 9.The group of interviewees was composed of HCWs from Médecins sans Frontières (Spain, Belgium, and Switzerland), Centers for Disease Control and Prevention (USA), WHO (Headquarters, Switzerland and Nigeria), Robert Koch-Institut (Germany), Health and Safety Executive (UK), Health Protection Agency (UK), Institute of Tropical Medicine Antwerp (Belgium), Leiden University Medical Center (The Netherlands) and independent nurses/clinicians. The views expressed in the interviews are views of individuals and not of their respective organisations.

[10] Sagripanti J.L., Lytle C.D. (2011). Sensitivity to ultraviolet radiation of Lassa, vaccinia, and Ebola viruses dried on surfaces. Arch. Virol..

[11] Nalgene® Polyolefin Plastic Paper Sheets. Supplier: Thermo Scientific [Internet]. https://us.vwr.com/store/catalog/product.jsp?catalog_number=51280-114.

[12] Bente D.A., Friesen J., White K., Koll J., Kobinger G.P. (2011). A computerized data-capture system for animal biosafety level 4 laboratories. J. Am. Assoc. Lab. Anim. Sci..

[13] Bausch D.G., Towner J.S., Dowell S.F., Kaducu F., Lukwiya M., Sanchez A., Nichil S.T., Ksiazek T.G., Rollin P.E. (2007). Assessment of the risk of Ebola virus transmission from bodily fluids and fomites. J. Infect. Dis..

