# Supplementary material for: Clinical Documentation and Data Transfer from Ebola and Marburg Virus Disease Wards in Outbreak Settings: Health Care Workers’ Experiences and Preferences
Source: Viruses. 2014 Feb 19;6(2):927–37. doi: 10.3390/v6020927 (PMC3939489; doi:10.3390/v6020927)
Supplement: Supplementary File 1 — Supplementary Information (PDF, 183 KB) [file viruses-06-00927-s001.pdf]

Topic guide for Semi-Structured Interviews

GENERAL INFORMATION ABOUT THE INTERVIEWEE:

Name:

Current Affiliation:

Name of current position;

Email:

Telephone:

Been to an outbreak yes no

Worked on field isolation ward yes no

If yes in which function

Has worked on isolation ward in developed country yes no

If yes in which function?

Which isolation approach was followed on the ward? Patient isolator or protective gear for staff?

TOPIC 1: REASONS FOR LOSS OF DATA

1. Did clinical data get documented?

2. If not – what do you think are the obstacles?

3. Did data get transferred from isolation wards?

4. If not – what do you think are the obstacles?

5. Do you think documentation and safe transfer of clinical data has been a priority? Why?

TOPIC 2: METHODS OF DATA EXTRACTION

6. What methods of data transfer out of isolation wards have you seen in practice or are you aware of?

7. Who in the team (which function) was responsible for data transfer?

TOPIC 3: ADVANTAGES/ DISADVANTAGES OF THE METHOD

8. Can you name any disadvantages of the method you have seen in use?

9. Can you name any advantages of the method?

10. How much time did it take to transfer clinical patient data out of isolation wards per day/per patient?

11. Do you have any concerns about the practicability of this method?

12. Do you have any safety concerns about the method of data collection?

13. How did you secure confidentiality when transferring clinical data?

14. Did you have any problems with technology?

TOPIC 4: IMPROVEMENTS OF METHODS

15. Can you think of any improvements to the methods in use?

16. Have any improvements been implemented?

TOPIC 5: NEW METHODS

17. Can you think of any new methods that could work better?
18. Why would the proposed method work better?
    -Does it save time?
    -Is it safer?
    -Is it more acceptable to the community?
    -Would it protect patient confidentiality in a better way?
    -Is it costly?
    -Would it allow for daily data extraction?
    -Can you think of any issues of practicability?

LAST TOPIC: ANYTHING ELSE THE INTERVIEWEE WOULD LIKE TO ADD

19. Is there anything important aspect we did not address in this interview?

20. Any further comments you would like to make?
